# Supplementary material for: Envelope-specific B-cell populations in African green monkeys chronically infected with simian immunodeficiency virus
Source: Nat Commun. 2016 Jul 6;7:12131. doi: 10.1038/ncomms12131 (PMC4935802; doi:10.1038/ncomms12131)
Supplement: Supplementary Information — Supplementary Figures 1-12 and Supplementary Tables 1-6 [file ncomms12131-s1.pdf]

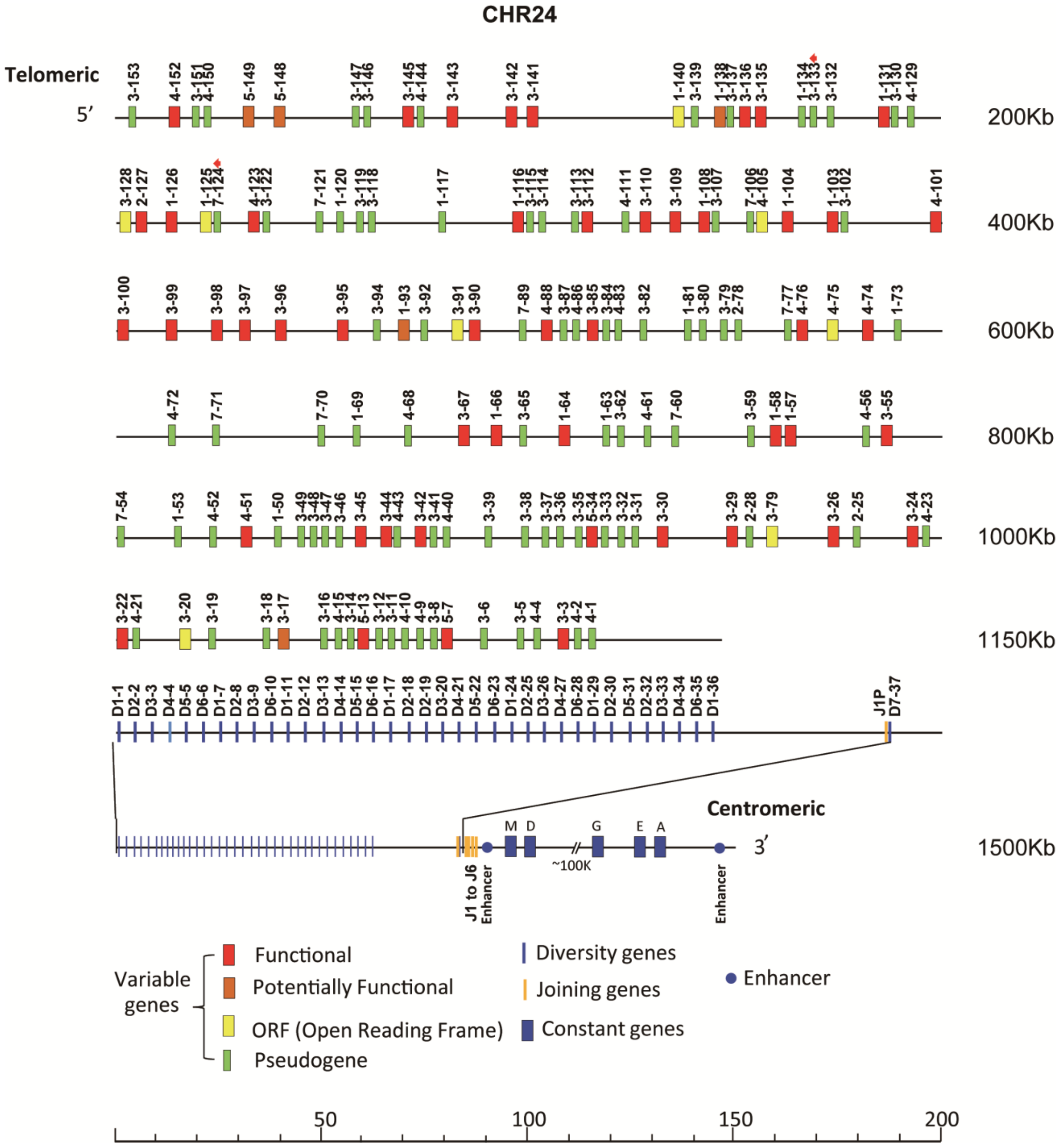

Supplementary Figure 1 Map of AGM (*Chlorocebus sabaeus*) immunoglobulin heavy chain locus on chromosome (CHR) 24. Representation of the identified functional and potentially functional, open reading frames, and pseudogenes of AGM V_H, D_H and J_H germline segments along with the Ig constant region genes of Ig M, D, G, A and E located on CHR24. Four additional functional AGM V_H gene segments that were identified from the unassembled contigs are not showed here (see Supplementary Data 1).

a

```
                                                         50                                                100
                                                          |                                                  |
HuIgG1   ASTKGPSVFPLAPSSKSTSGGTAALGCLVKDYFPEPVTVSWNSGALTSGVHTFPAVLQSSGLYSLSSVVTVPSSSLGTQTYICNVNHKPSNTKVDKKVE-
RhIgG1   ...............R...ES.......................S....................................V..............R..I
AGMIgG1  ...............R...ES.......................S....................................T..............R..I
HuIgG2   .............C.R...ES.....................................................NF.....T...D..........T..R
RhIgG2   ............SC.R...QS................................Q...........................V...V.E........T.GL
AGMIgG2  ----------------------------------------------------------------------------------------------------
HuIgG3   .............C.R.................................................................T..............R..L
HuIgG4   .............C.R...ES.........................................................K..T...D..........R..S
RhIgG3   ............SC.R...QS............................................................V...V.E........R..F
RhIgG4   ............S..R...ES............................................................V...V.E........R..F

                                                                                                           200
                                          Hinge region                                                       |
HuIgG1   ----------------------------------------------PKSCDKTHTCPPCPAPELLGGPSVFLFPPKPKDTLMISRTPEVTCVVVDVSHED
RhIgG1   KTCG--------------------------------------------GGS.PP...........................................Q..
AGMIgG1  KTCG--------------------------------------------GGG.PP...............................N...........Q..
HuIgG2   -----------------------------------------------.C.---VE......-PVA..................................
RhIgG2   PCR--------------------------------------------------S.......-...................................Q.E
AGMIgG2  --------------------------------------------------------------...................................Q.E
HuIgG3   KTPLGDTTHTCPRCPEPKSCDTPPPCPRCPEPKSCDTPPPCPRCPE.....TPPP..R..........................................
HuIgG4   -----------------------------------------------.YG---PP..S.....F.................................Q..
RhIgG3   TPPC--------------------------------------------GDTT-PP..........................................Q..
RhIgG4   TP----------------------------------------------------P..........................................Q..

                                                         250                                               300
                                                          |                                                  |
HuIgG1   PEVKFNWYVDGVEVHNAKTKPREEQYNSTYRVVSVLTVLHQDWLNGKEYKCKVSNKALPAPIEKTISKAKGQPREPQVYTLPPSRDELTKNQVSLTCLVK
RhIgG1   .D.......N.A...H.Q.....T..............T..........T............Q.....D................E..............
AGMIgG1  ...Q.............Q.....T..............R..........T............Q.....D............A...E..............
HuIgG2   ...Q.....................F...F........V.................G...........T................E.M............
RhIgG2   .D...............Q.......F............T..........T...........RQ..V..T..............P.E..............
AGMIgG2  ...Q.............Q.......N............T..........T...........RQ..V..T..............P.E............I.
HuIgG3   ...Q.K.......................F......................................T................E.M............
HuIgG4   ...Q.....................F..............................G..SS.......................QE.M............
RhIgG3   ...Q.......A...H.Q.....R.F............T..........T......G......................I...PQE.............T
RhIgG4   ...Q.............Q.....R.F............T..........T......G......................I...PQE.............T

                                                         350
                                                          |
HuIgG1    GFYPSDIAVEWESNGQPENNYKTTPPVLDSDGSFFLYSKLTVDKSRWQQGNVFSCSVMHEALHNHYTQKSLSLSPGK
RhIgG1    .......V.....S.....T.............Y..................................---------
AGMIgG1   .......V...........T.............Y.................T....................V....
HuIgG2    ..........................M..................................................
RhIgG2    .......V...A.......T.............Y.................T................---------
AGMIgG2   .......V......R....T.............Y.................T....................V....
HuIgG3    .............S.......N....M........................I............RF...........
HuIgG4    ......................................R.........E.........................L..
RhIgG3    ...................T.............Y.................T................---------
RhIgG4    ...................T.............YL.......N.....P..I.T..............---------
```

b

```
                                                        50                                                 100
                                                        |                                                    |
HuIgA1       SPTSPKVFPLSLCSTQPDGNVVIACLVQGFFPQEPLSVTWSESGQGVTARNFPPSQDASGDLYTTSSQLTLPATQCLAGKSVTCHVKHYTNPSQDVTVPC
HuIgA2       ............D..PQ.....V......................N..............................PD......................
RMIgA_AI     ...K........EG..S.~...V..........K..N....K..A...VI....R.....G............A..P.SE......E.....R...A...
RMIgA_AII    ...R........EG..S.~...V.............N....K..A...VI....R.....G............A..P.SE......E.....R...G...
RMIgA_BI     ...R......N.....S.~...V.............N....K..A...VI..........G............A..P.SE......E.........A...
RMIgA_BII    ...R............S.~.................N....K..A...VI....R.....G............A..P.SE......E.........A...
RMIgA_CI     ...R......N.....S.~...V.............N....K..AD..VI..........G............A..P.SE......E.........A...
RMIgA_CII    ...R........EG..S.~...V.............N....K..A...VI..........G............A..P.SE......E....AR...A...
RMIgA_DI     ...R........EG..S.~...V.............N....K..A...VI....R.....G............A..P.SE......E.....R...G...
RMIgA_DII    ...R........EG..S.~...V.............N....K..A...VI..........G............A..P.SE......E....AR...A...
RMIGA_RJZ7   ............KG..S.....V.............N...NK..A...VI..........G...............P.TE......E.........A.R.
RMIGA_RUA8   ..........N.....S.~...V.............N....K..AD..VI..........G............A..P.SE......E.........A...
AGMIGA       ............EG..S.....V.............N....KN.E~~~VI......A...G............A..P.SE....R.E.........A...

                                                       150                                                 200
                Hinge region                            |                                                    |
HuIgA1       PVPSTPPTPSPSTPPTPSPSCCHPRLSLHRPALEDLLLGSEANLTCTLTGLRDASGVTFTWTPSSGKSAVQGPPERDLCGCYSVSSVLPGCAEPWNHGKT
HuIgA2       ...-------------.P.P....................................A...................................Q.....E.
RMIgA_AI     R~VPP.KC..--------LK.DK.....R........................P..A..........N...QS..H.P..................NRV.
RMIgA_AII    R~VPP.NCR----------L.DK.....R........................P..A..A.......N...QS..H.P..................NRV.
RMIgA_BI     VP.I...C.----------...E.....R......................K.P..A..........N...QS.K..P..................NRE.
RMIgA_BII    V~.QPK.CL-----------.DK.....R........................P..A..........N...QS..H.P..................NRV.
RMIgA_CI     VP.I...C.----------...E.....R........................P..A..........N...QS.K..P..................NRE.
RMIgA_CII    V~SQ.K.CL-----------.DE.....R........................P..A..........N...QS.K..P..................NRV.
RMIgA_DI     R~VPP.NCR----------L.DK.....R........................P..A..A.......N...QS..H.P..................NRV.
RMIgA_DII    V~SQ.K.CL-----------.DE.....R........................P..A..........N...QS.K..P..................NRV.
RMIGA_RJZ7   RD.QPK.CL-----------.DK.Q...R........................P..A..A.......N...QS..H.P..................NRE.
RMIGA_RUA8   VP.I...C.----------...E.....R......................K.P..A..........N...QS.K..P..................NRE.
AGMIGA       V~..PKTCF-----------.DE.....R........................P..A..........N...QS..H.P..................N.V.

                                                       250                                                 300
                                                        |                                                    |
HuIgA1       FTCTAAYPESKTPLTATLSKSGNTFRPEVHLLPPSEELALNELVTLTCLARGFSPKDVLVRWLQGSQELPREKYLTWASRQEPSQGTTTFAVTSILRVA
HuIgA2       ......H..L......NIT.................................................................................
RMIgA_AI     .....EH..LE.Q....I......................................E.......K.TEQ...D.....E..K..................
RMIgA_AII    .N...NH..L.......I......................................E.......K.TEQV..D.....E..K..................
RMIgA_BI     .....NH..LE......I......................................E.......K.TEQ...D.....E..K..................
RMIgA_BII    .....EH.QL.......I......................................E.......K.TEQ...D.....E..K..................
RMIgA_CI     .....NH..LE......I......................................E.......K.TEQ...D.....E..K..................
RMIgA_CII    ...K.EH..LE......I......................................E.......K.TEQ...D.....E..K..................
RMIgA_DI     .N...NH..L.......I......................................E.......K.TEQV..D.....E..K..................
RMIgA_DII    ...K.EH..LE......I......................................E.......K.TEQ...D.....E..K..................
RMIGA_RJZ7   .....NH..L.......I......................................E.......K.TEQ...D.....E..K..................
RMIGA_RUA8   .....NH..LE......I......................................E.......K.TEQ...D.....E.....................
AGMIGA       .....NH..LE.Q....IT.....................................E.......K.TEQ...D.....E..K.....P............

                                                       350
                                                        |
HuIgA1       AEDWKKGDTFSCMVGHEALPLAFTQKTIDRLAGKPTHVNVSVVMAEVDGTCY
HuIgA2       ....................................................
RMIgA_AI     ..........................................----------
RMIgA_AII    ..........................................----------
RMIgA_BI     ..........................................----------
RMIgA_BII    ..........................................----------
RMIgA_CI     ..........................................----------
RMIgA_CII    ..........................................----------
RMIgA_DI     ..........................................----------
RMIgA_DII    ..........................................----------
RMIGA_RJZ7   ..........................................----------
RMIGA_RUA8   ..........................................----------
AGMIGA       .................................-------------------
```

c

```
                                                                50                                                100
                                                                 |                                                  |
HuIgD    APTKAPDVFPIISGCRHPKDNSPVVLACLITGYHPTSVTVTWYMGTQSQPQRTFPEIQRRDSYYMTSSQLS--TPLQQWRQGEYKCVVQHTASKSKKE-I
RhIgD    -----A.......A.QL................N.K......HL...I.N.IM...TE.EG.-.T.....APTP..S.QH......T.K..P.DTS..KT
AGMIgD   .S.......-.T.A.EL................N.K......HL...I.N.IM...TDTQG.-.T.....V-TP..S.RH......T.K..P.NTN..KT

                                                               150                                                200
                                                                 |                                                  |
HuIgD    FRWPESPKAQASSVPTAQPQAEGSLAKATTAPATTRNTGRGGEEKKKEKEKEEQEERETKTPECPSHTQPLGVYLLTPAVQDLWLRDKATFTCFVVGSDL
RhIgD    ..........YP....V......G.S...RP...N.....---RE.EDE.EK..Q.G...................P..L....FQ..V...........
AGMIgD   .H........YP..S........G.S...RS...N.....---RE.ED..EK..Q.G........R......I...P..L....F...............

                                                               250                                                300
                                                                 |                                                  |
HuIgD    KDAHLTWEVAGKVPTGGVEEGLLERHSNGSQSQHSRLTLPRSLWNAGTSVTCTLNHPSLPPQRLMALREPAAQAPVKLSLNLLASSDPPEAASWLLCEVS
RhIgD    Q....S........K..M...P..Q............A......................S.K.............R.......................
AGMIgD   Q....S........K.DM...P..Q............A......................S.K.............R.......................

                                                               250
                                                                 |
HuIgD    GFSPPNILLMWLEDQREVNTSGFAPARPPPQPRSTTFWAWSVLRVPAPPSPQPATYTCVVSHEDSRTLLNASRSLEVSYVTDHGPMK
RhIgD    D............N.......W..TTH.T...G..M..........G.T.............................---------
AGMIgD   D.................S.W..TTH.T...G..M..........G.T.....................V.YLTDRGP.KX-----
```

d

```
                                                                50                                                100
                                                                 |                                                  |
HuIgM    GSASAPTLFPLVSCENSPSDTSSVAVGCLAQDFLPDSITLSWKYKNNSDISS-TRGFPSVLRGGKYAATSQVLLPSKDVMQGTDEHVVCKVQHPNGNKEK
RhIgM    ................A.L..NE................F...F....N..KGVW...................A........................Q
AGMIgM   ................ALL..NK................F...F.......KGVW........D..V.......A.T......................Q

                                                               150                                                200
                                                                 |                                                  |
HuIgM    NVPLPVIAELPPKVSVFVPPRDGFFGNPRKSKLICQATGFSPRQIQVSWLREGKQVGSGVTTDQVQAEAKESGPTTYKVTSTLTIKESDWLGQSMFTCRV
RhIgM    ......L..R..N...........V....E...............E.............I...R.E..........F.......VS.R...S..V.....
AGMIgM   ......V..Q..N....I......V....E...............E.............I.....E..........F.......VS.R...S..V.....

                                                               250                                                300
                                                                 |                                                  |
HuIgM    DHRGLTFQQNASSMCVPDQDTAIRVFAIPPSFASIFLTKSTKLTCLVTDLTTYDSVTISWTRQNGEAVKTHTNISESHPNATFSAVGEASICEDDWNSGE
RhIgM    ........K.V..V.G.NP...............................A.......T........L............G...................
AGMIgM   ........K.V..V.G.NP.I.............................A................L............G...................

                                                               350                                                400
                                                                 |                                                  |
HuIgM    RFTCTVTHTDLPSPLKQTISRPKGVALHRPDVYLLPPAREQLNLRESATITCLVTGFSPADVFVQWMQRGQPLSPEKYVTSAPMPEPQAPGRYFAHSILT
RhIgM    ..R.......................M..................................I......................................
AGMIgM   W.R......................PM..................................I......................................

                               430
                                |
HuIgM    VSEEEWNTGETYTC-VAHEALPNRVTERTVDKST
RhIgM    ....D.........V...................
AGMIgM   ....D.........V...................
```

e

```
                                                          50                                               100
                                                           |                                                 |
HuIgE     ASTQSPSVFPLTRCCKNIPSNATSVTLGCLATGYFPEPVMVTCDTGSLNGTTMTLPATTLTLSGHYATISLLTVSGAWAKQMFTCRVAHTPSSTDWVDNK
RhIgE     ..I...F....IP...H.A.......................W.A....RS........F.P..................ET...H.V.....A.KEV..
AGMIgE    ..I...F....IP...H.A.D.....................W.A....RS........F.P..................ET...H.V.....A.KEV..

                                                         150                                               200
                                                           |                                                 |
HuIgE     TFSVCSRDFTPPTVKILQSSCDGGGHFPPTIQLLCLVSGYTPGTINITWLEDGQVMDVDLSTASTTQEGELASTQSELTLSQKHWLSDRTYTCQVTYQGH
RhIgE     ..G....N..............DD............I......A..V....N....K.NSP.PPA............F..A..................T
AGMIgE    ..G....N..............DD............I......A..V....N....E.NSP.PPA............F..P..................G

                                                         250                                               300
                                                           |                                                 |
HuIgE     TFEDSTKKCADSNPRGVSAYLSRPSPFDLFIRKSPTITCLVVDLAPSKGTVNLTWSRASGKPVNHSTRKEEKQRNGTLTVTSTLPVGTRDWIEGETYQCR
RhIgE     .YN............................S................E..............P.IPTT.K...........I...V.Q...........
AGMIgE    .Y.............................S................E..............PQVRP.R.R..........I...V.Q...........

                                                         350                                               400
                                                           |                                                 |
HuIgE     VTHPHLPRALMRSTTKTSGPRAAPEVYAFATPEWPGSRDKRTLACLIQNFMPEDISVQWLHNEVQLPDARHSTTQPRKTKGSGFFVFSRLEVTRAEWEQK
RhIgE     ..........V..M.............V.....KLE.........................SD.........V....................K......
AGMIgE    ..........V..M.............V.....KLE.........................SD.........V....................K......

                         430
                           |
HuIgE     DEFICRAVHEAASPSQTVQRAVSVNPGK-
RhIgE     ...............WI..Q........X
AGMIgE    ...............WI..Q.....L..X
```

Supplementary Figure 2 Sequence alignment of AGM, RM, and human Ig constant regions. Panel **a** shows sequence alignment of IgGs from the three species. The underlined text shows the hinge region of IgGs of three species. Sequences highlighted in yellow represent the identical residues in the hinge region between AGM IgG1 and RM IgG1. Sequences in red show the shared unique mutations in the C_H3 domain of AGM IgG2 and RM IgG2. Panel **b** shows sequence alignment of IgAs of three species. The hinge region of human IgA1 is highlighted in red. Ten RM intra-species heterogenetic IgA alleles were used in the alignment. The sequence alignments of IgD (**c**), IgM (**d**) and IgE (**e**) of three species are also represented.

```
                                                                         50                                                100
                                                                         |                  μE1                  μE5      |
AGM_IgH_Eμ      CATCATAAATCAAGTTTATTTTTTTAATTAATTGAGTGAAGCTGGGAGCGGATGATGAGTTAGAGTCAAGATGGCTGCATGGGGGTCTTGGGCACCCACA
Rhesus_IgH_Eμ   ..............................................................................G.........C..........G
Human_IgH_Eμ    ....................................C........A...A........A.............................CC..........

                                                                         150                                               200
                  μE2       μA        μE3                μB             |                                                  |
AGM_IgH_Eμ      GCAGGTGGCAGGAAGCAGGTCACCGCAAGAGTCTATTTTAGGAAGCAAAAAAACACAACTGGTAGATTTATCACTTCTGGTTGTGAAGAGGTGGTTTCGC
Rhesus_IgH_Eμ   .........G......................T...... .....-.....C...G.T....A...G..............................
Human_IgH_Eμ    ..........................G...............................T....A................................T..

                                                                                                                   300
                                                  μE4    Octamer motif                                             |
AGM_IgH_Eμ      CTGGGCCCAGATCTGAAAGTGCTCTACTGAGCAAAACAACACCTGGACGATTTGCGTTTCTAAAATAAGGCGAGGCTGACCGAAACTGGAAAGGCTC-TT
Rhesus_IgH_Eμ   .......................C.............. ..............................A........................T..
Human_IgH_Eμ    .CA........................................A.......................................A.......----

                                                                       350
                                        CACA Box                       |                TATA Box
AGM_IgH_Eμ      TTTTTTAACTATCCGAATTTCATTTCCAATCTTAGCTTATCAACTGCTAGTTTGTGCAAACAGGATATCAACTTCTAAACTGCATTCATTTT
Rhesus_IgH_Eμ   .............A.........C.............. .............................................C.......
Human_IgH_Eμ    .............T.................................................C............................
```

Supplementary Figure 3 Comparison of IgH intronic enhancer core region (cEμ) of AGM, RM and human. The **c**Eμ elements, Octamer motif, CACA box, and TATA box were highlighted and underlined. RM cEμ intronic enhancer region was identified from the assembly on Oct. 2010 (BGI CR_1.0/rheMac3).

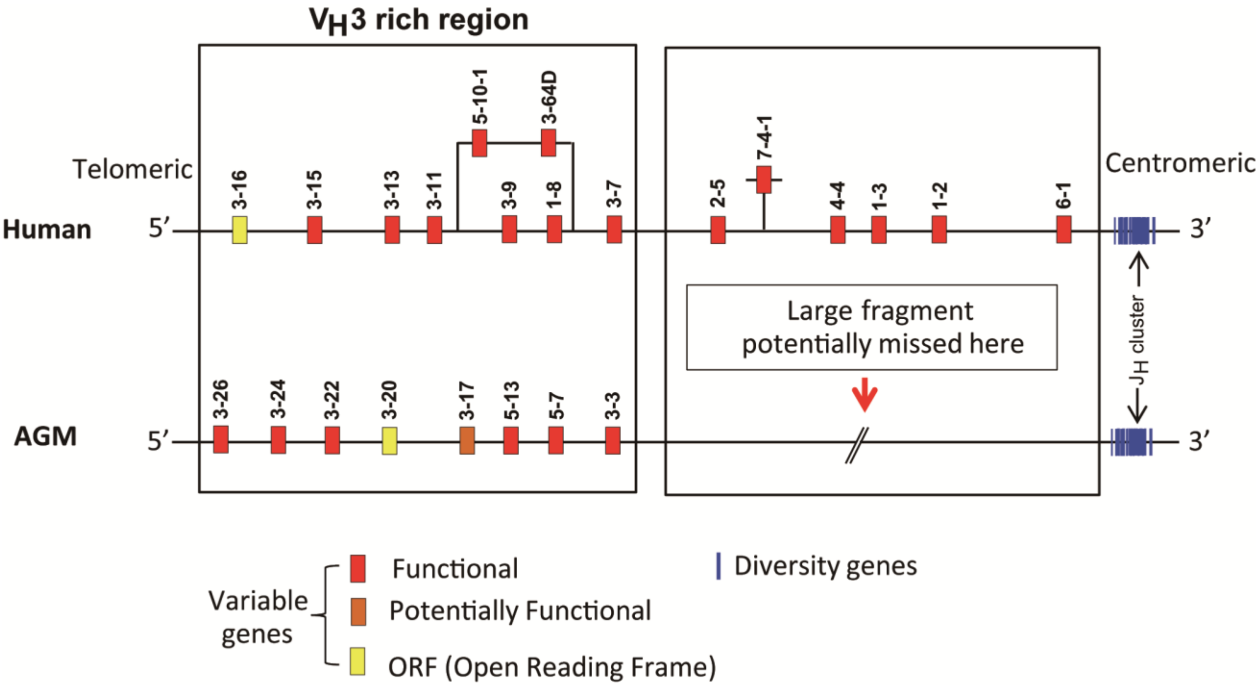

Supplementary Figure 4 Comparison of the centromeric region of V_H locus in AGM and human genomes. Mapping of AGM VH gene segments in reference of human genome indicated that a large DNA fragment is potentially missing in the cerntromeric side of AGM V_H locus, in which VH6, VH7 and VH2 might be located by comparing human V_H locus.

a

Family	AGM	RM	HU
Vκ1	25	25	15
Vκ2	24	22	8
Vκ3	10	9	7
Vκ4	1	1	1
Vκ5	1	2	1
Vκ6	2	2	2
Vκ7	1	1	0
Sum	64	62	34

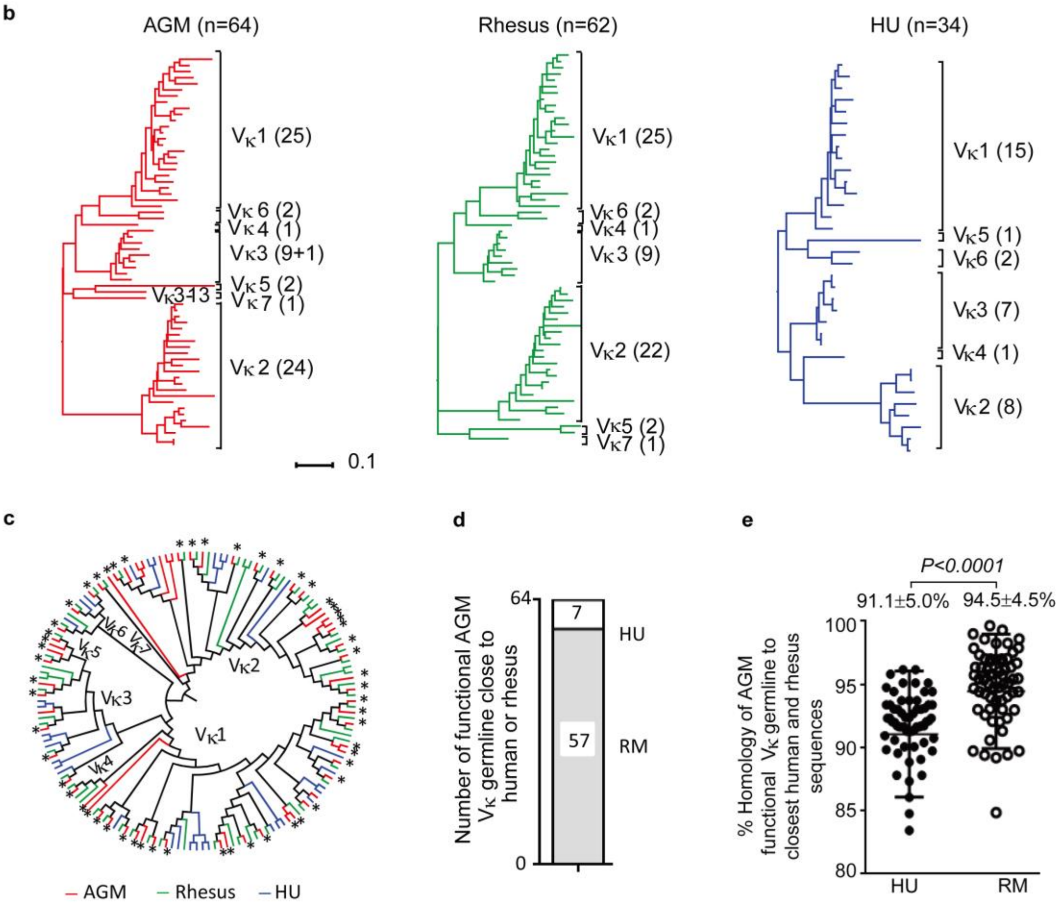

Supplementary Figure 5 AGM, RM and human immunoglobulin kappa chain variable gene (V_κ) homology. AGM V_κ genes are genetically more similar to those of RM than human. **Panel a** shows numbers of functional V_κ genes from each species. **Panel b** shows phylogenetic comparison of V_κ families of AGM, RM and human. AGM V_κ 3-13 is located out of V_κ 3 family branch and has a human homologue gene as IGKV3-NL5*01 F. **Panel c** shows joint radial phylogenic tree of AGM (red lines), RM (green lines), and human (blue lines) V_κ genes; the AGM V_κ genes that are genetically more similar to RM than human V_κ homologs are indicated with star (*). **Panel d** indicates the number of AGM V_κ genes that are genetically more similar to RM (grey box) or human (white box) than AGM. **Panel e** indicates percent homology of AGM V_κ genes to those of RM and human. Percent homology between V_κ germline genes between AGMs, humans and RMs were compared ($p<0.0001$) using an Exact Wilcoxon rank sum test. The data in Panel e shows means±sd.

a

Family	AGM	RM	HU
Vλ1	8	10	5
Vλ2	7	7	5
Vλ3	13	14	10
Vλ4	2	2	3
Vλ5	7	6	4
Vλ6	1	3	1
Vλ7	5	4	2
Vλ8	0	1	1
Vλ9	1	1	1
Vλ10	1	1	1
Vλ11	0	1	0
Sum	45	51	33

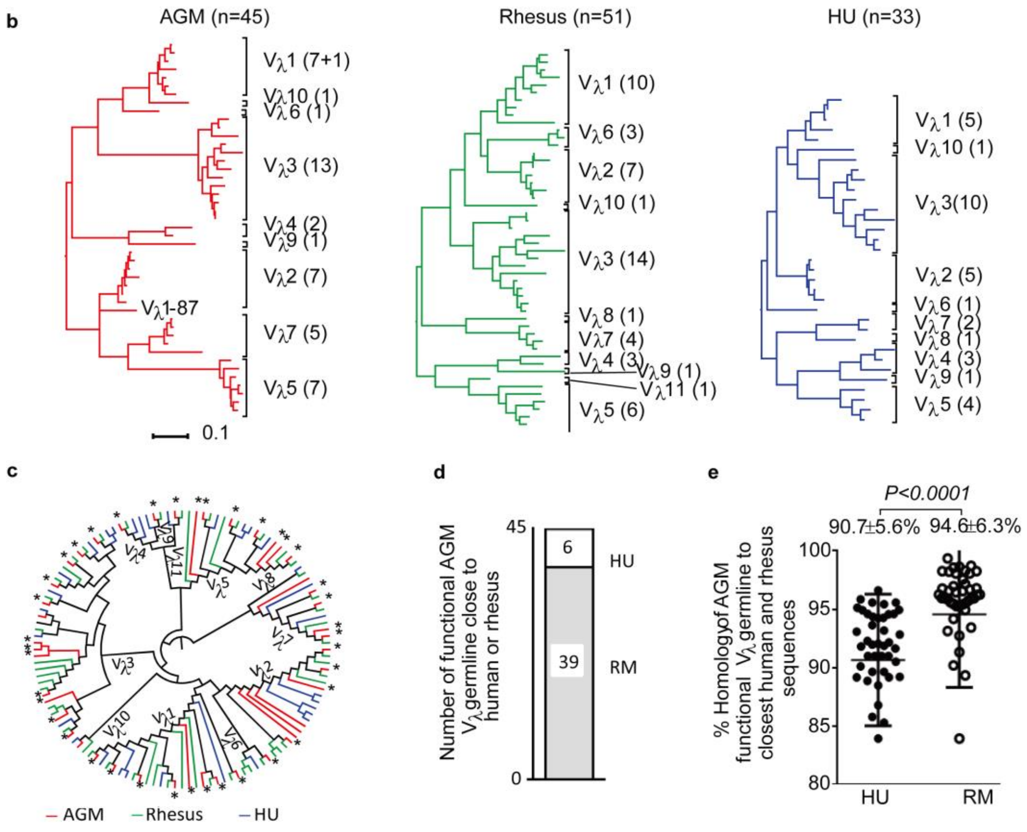

Supplementary Figure 6 AGM, RM and human immunoglobulin lambda chain variable gene (V_λ) homology. AGM V_λ genes are genetically more similar to those of RM than human. **Panel a** shows number of functional V_λ genes in each species. **Panel b** shows phylogenetic comparison of AGM, RM and human V_λ families. AGMLV1-87 is located out of V_λ1 family branch, and has the human homologue gene as IGLV1-62*01 P. **Panel c** shows joint radial phylogenic tree of AGM (red lines), RM (green lines), and human (blue lines) V_L genes; the AGM V_λ genes that are genetically more similar to RM than human V_λ homologs are indicated with star (*). **Panel d** shows number of AGM V_λ genes genetically more similar to RM (grey box) or human (white box) than AGM. **Panel e** shows percent homology of AGM V_λ genes to those of RM and human. Percent homology between V_λ germline genes between AGMs, humans and RMs were compared ($p<0.0001$) using an Exact Wilcoxon rank sum test. The data in Panel e shows means±sd.

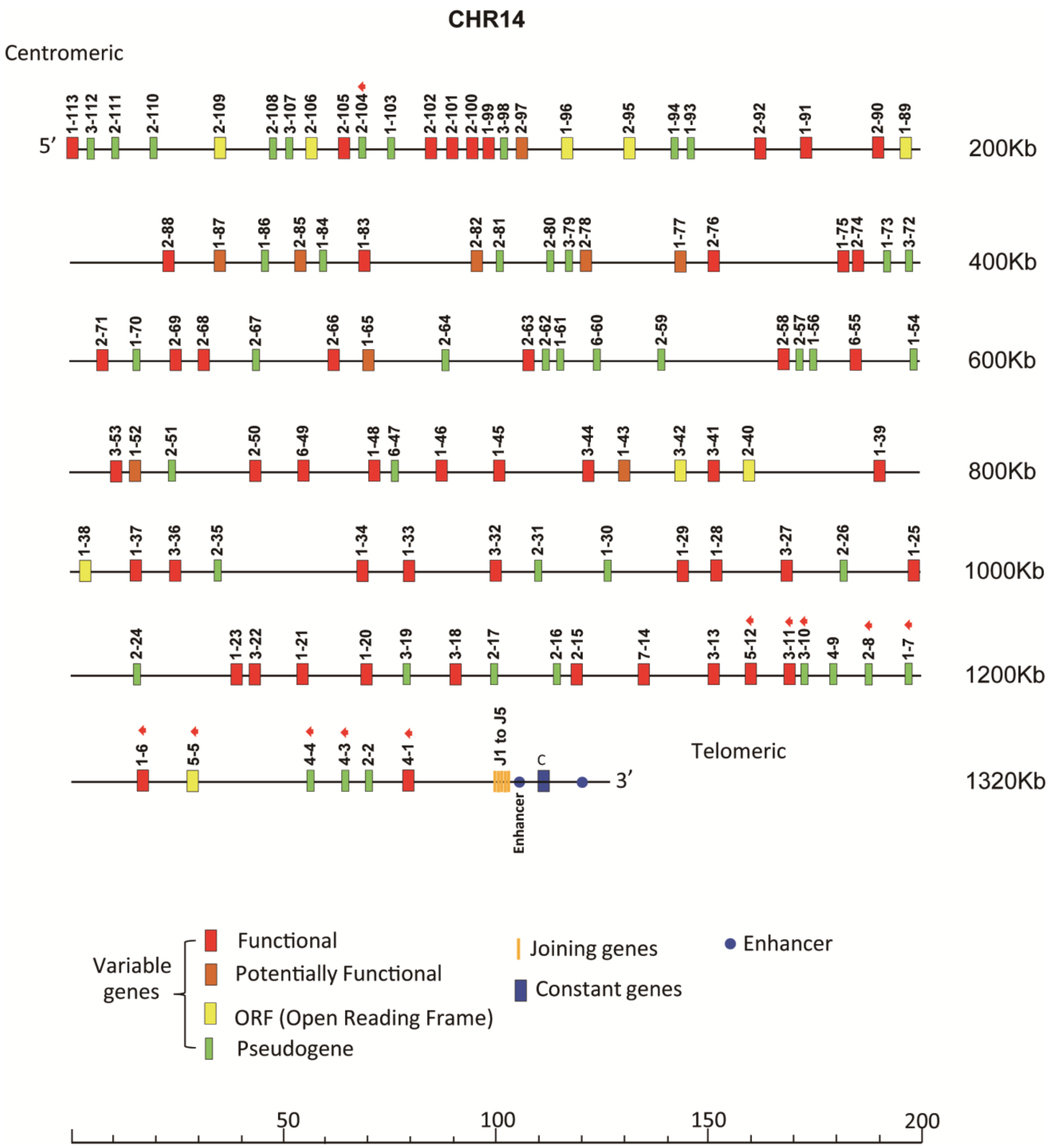

Supplementary Figure 7 Map of AGM (*Chlorocebus sabaeus*) immunoglobulin κ locus on chromosome (CHR) 14. Shown are the identified functional, potentially functional, open reading frames, and pseudogenes of AGM V_κ and J_κ germline segments along with the Ig kappa chain constant region gene located on CHR14.

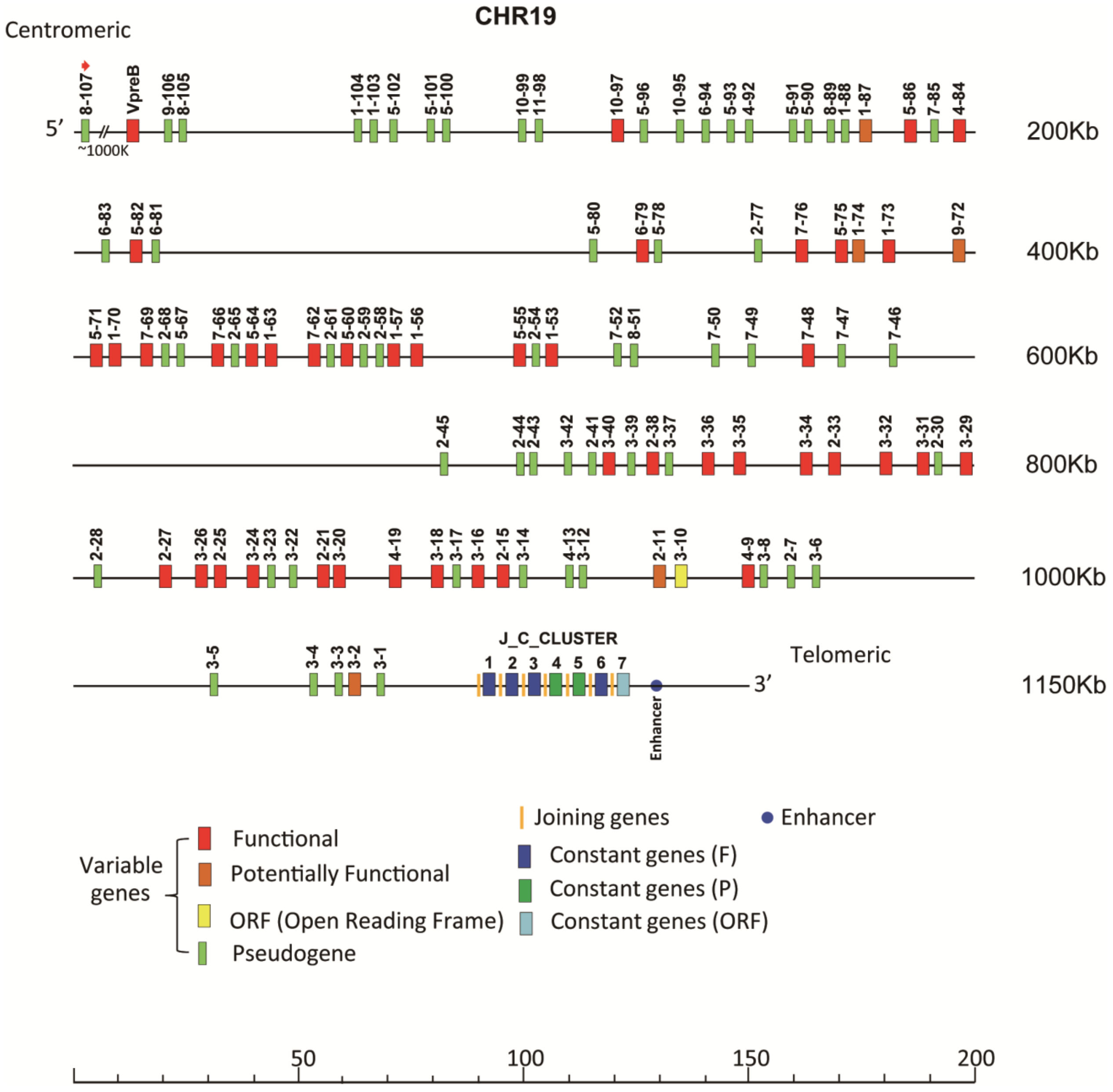

Supplementary Figure 8 Map of AGM (*Chlorocebus sabaeus*) immunoglobulin λ locus on chromosome (CHR) 19. Shown are the identified functional, potentially functional, open reading frames, and pseudogenes of AGM V_λ and J_λ germline segments along with the Ig lambda chain constant region gene located on CHR19.

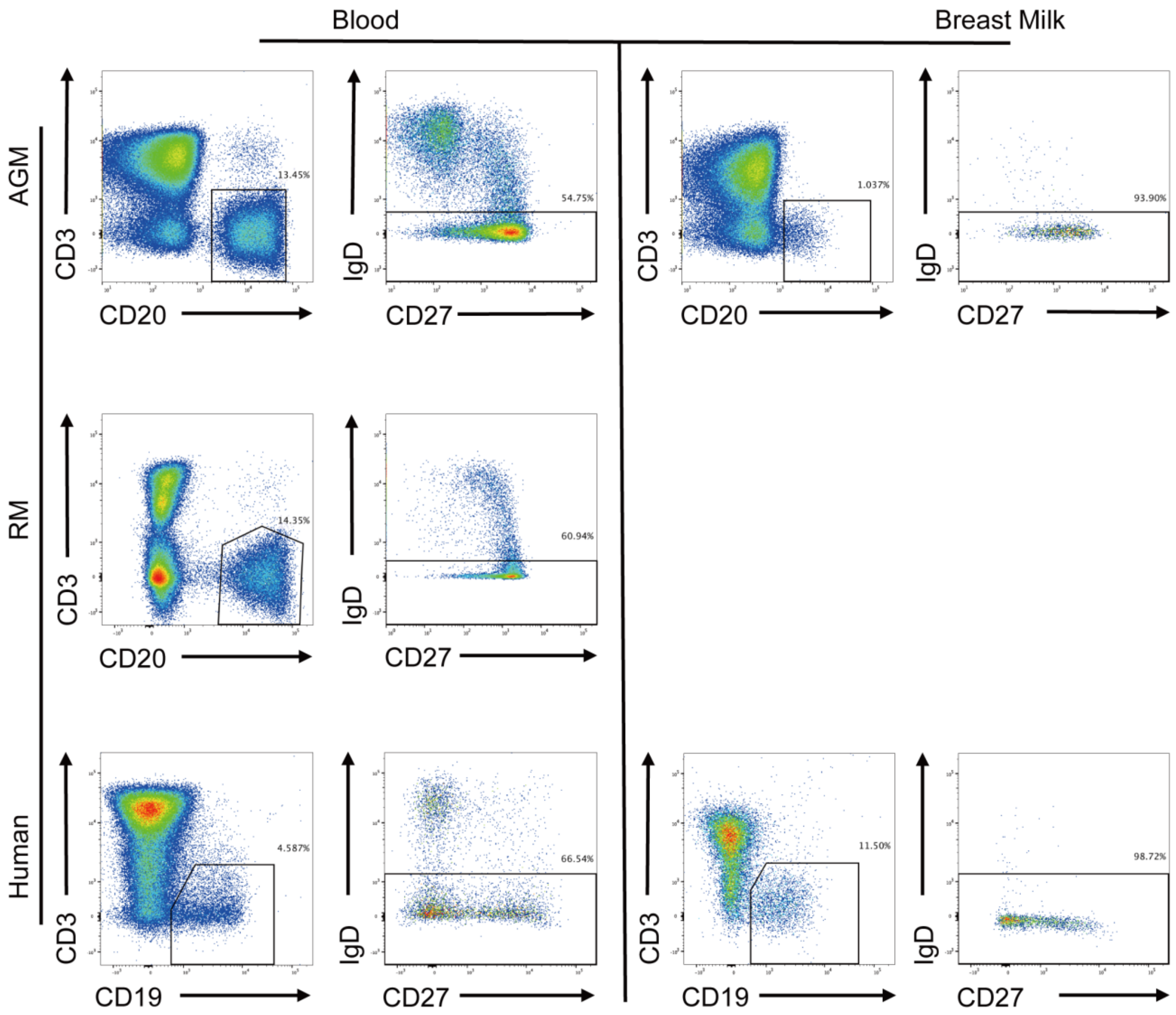

Supplementary Figure 9 Gating strategy for total memory B cell populations in blood and milk of SIV/HIV-infected, AGM, RM, and human. Total memory B cells of SIV-infected AGM and RM were gated as $CD3^-/CD20^+/IgD^-$ (top two rows). Total memory B cells from blood and milk of HIV-infected human were gated as $CD3^-/CD19^+/IgD^-$ (bottom row).

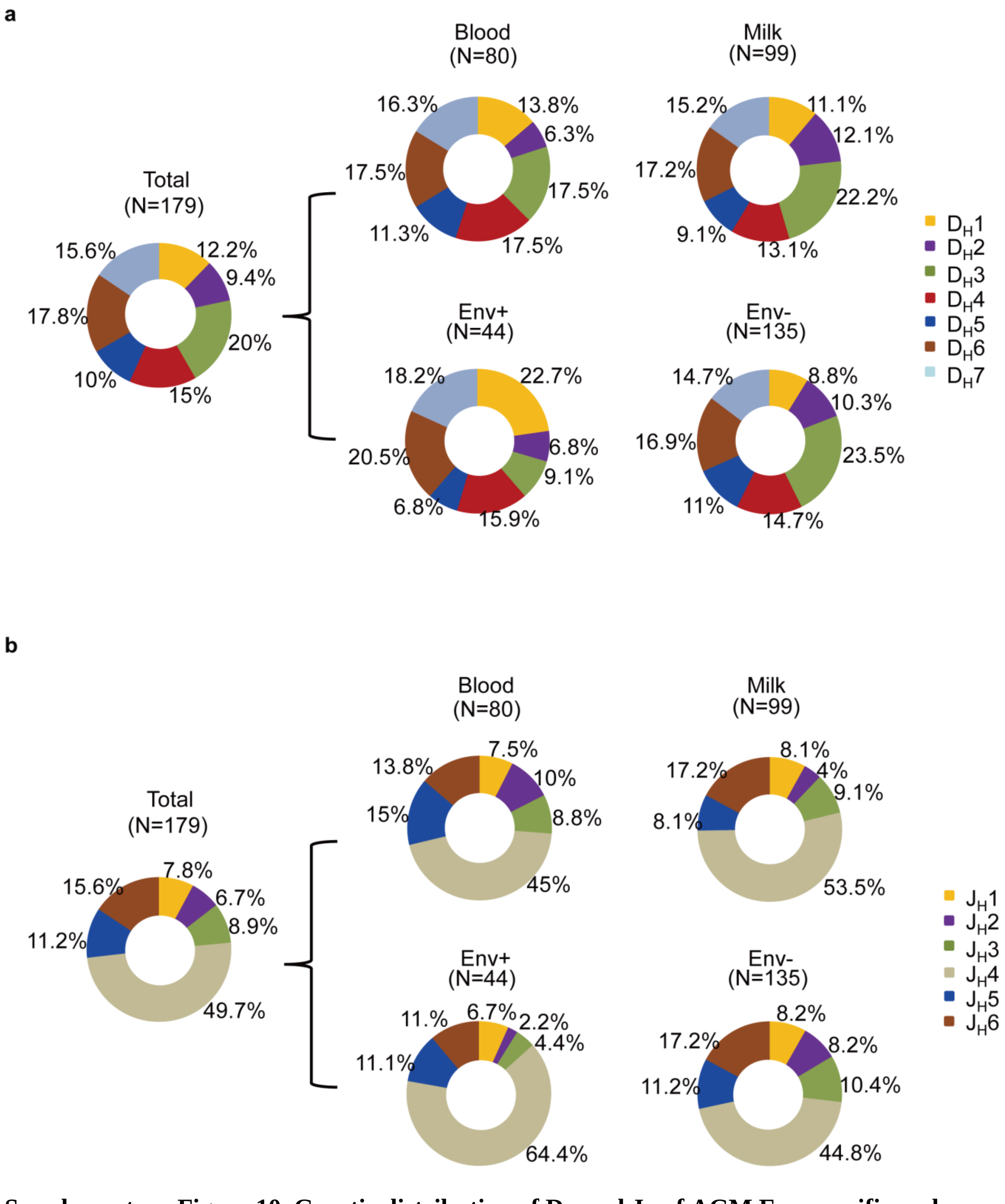

Supplementary Figure 10 Genetic distribution of D_H and J_H of AGM Env-specific and non Env-specific mAbs. The distribution of D_H gene families (Panel **a**) and J_H gene families (Panel **b**) isolated from AGM blood and milk samples.

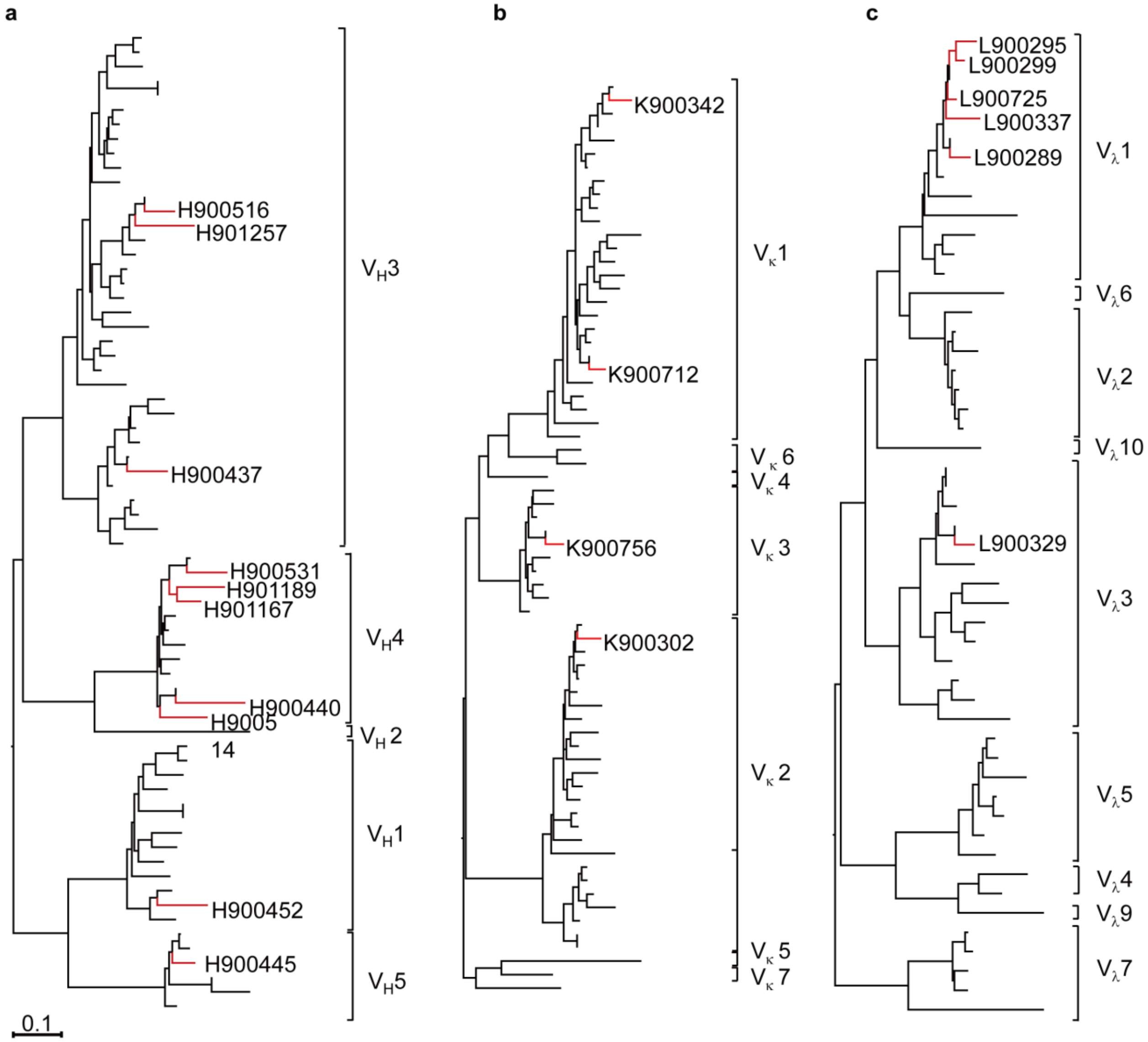

Supplementary Figure 11 The phylogenetic relationship of 10 purified SIV Env-reactive mAbs to AGM heavy and light chain variable germline genes. The phylogenetic relationship of V_H (Panel **a**), V_κ (Panel **b**) and V_λ (Panel **c**) genes of 10 purified SIV Env-reactive mAbs to AGM V_H germline genes.

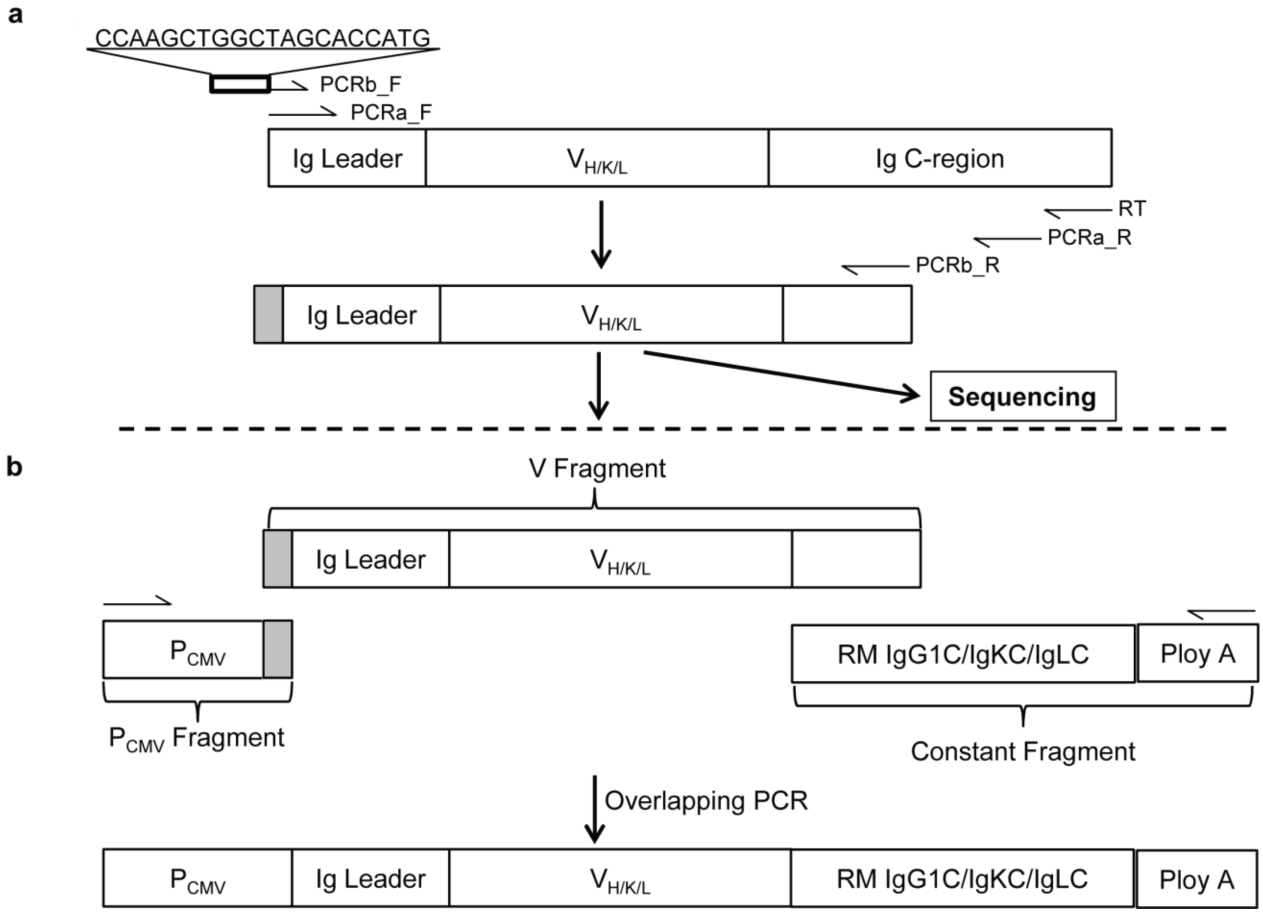

Supplementary Figure 12 Schematic diagram for the generation of linear full-length Ig heavy- and light-chain gene constructs. (**a**) Schematic representation of cloning rhesus immunoglobulin heavy and light chain variable genes by semi-nest PCR. (**b**) The Ig H/L PCR products were sequenced and the functional genes were then assembled into full-length linear expression cassettes with CMV promoter and BGH Poly A by overlapping PCR.

Supplementary Table 1 Demographic, virologic, and immunologic characterization of HIV-1-infected individuals used for antibody isolation from blood and breast milk

#	Patient ID	Number of Antibodies Isolated			Patient Characteristics						
		Blood	Milk	Total	Age (yrs)	Sex	Country of Origin	Specimen Draw Date	Viral Load (RNA copies/mL)	CD4 count (cells/mm[1])	ARV
1	711-12-071-8	93	0	93	21	M	USA	24-Jun-10	<40	528	Yes
2	700-12-051-1	68	0	68	59	M	USA	9-Oct-09	16,800	957	No
3	714-90-060-8	22	0	22	41	F	Malawi	3-Dec-07	<400	275	Yes
4	715-90-060-5	0	3	3	28	F	Malawi	2-Apr-08	32,164	519	No
5	714-09-360-1	0	31	31	18	F	Malawi	10-Mar-08	60,335	452	No
6	715-90-030-1	6	5	11	32	F	Malawi	26-Mar-08	75,934	406	No
7	715-90-120-9	14	2	16	25	F	Malawi	18-Jun-08	174,976	153	No

Antibodies Isolated		
	Blood	203
	Milk	41
	Total	244

[1] VL and CD4 count performed at the time of PBMC/milk cell isolation

Supplementary Table 2 Amino acid sequences of the upper, core and lower hinge of IGG molecules from AGM (*Chlorocebus Sabaeus*), RM, and human.

	Upper Hinge	Core	Lower Hinge
HU_IgG1	EPKSCDKTHT	CPPC	PAPELLGGP
RM_IgG1	EIKTCGGGSKPPT	CPPC	PAPELLGGP
AGM_IgG1	EIKTCGGGGKPPT	CPPC	PAPELLGGP
HU_IgG2	ERK	CCVECPPC	PAPPVAGP
RM_IgG2	GLP	CRSTCPPC	PAELLGGP
AGM_IgG2	not available	not available	ELLGGP (partial)
HU_IgG3	ELKTPLGDTTHT	CPRCP<br>EPKSCDTPPPCPRCP<br>EPKSCDTPPPCPRCP<br>EPKSCDTPPPCPRCP	APELLGGP
RM_IgG3	EFTPPCST	CPPC	PAPELLGGP
HU_IgG4	ESKYGPP	CPSC	PAPEFLGGP
RM_IgG4	EFTPP	CPPC	PAPELLGGP

Supplementary Table 3 Primer list for amplifying both AGM and rhesus macaque Ig pairs

Primers	Sequences	Tm
RT primers		
G_RT	TCTTGTCCACCTTGGTGTTG	51.8
M-RT	TGAATTTCCAGGAGAAAGTGATG	51.7
A_RT	TGTTTCCRGATTTTGAGATGGTG	53.5
D_RT	AAGGTCTTCTCCTTGCTTGTATC	53.5
E_RT	TTGGAATCTGCACACTTCTTG	50.5
CK_RT	TCTCTGGGATAGAAGTTATTCAG	51.7
CL_RT	CTTGTTGTTGCTCTGTTTGGAG	53
PCR1 primers		
IgH primers		
VH1+7_EXT1	CACCATGGACTGGACCTGGAGGMTCCTC	67.9
VH1+7_EXT2	CACCATGGACCTGACCCGGAGGATCCTTTTC	72.4
VH2_EXT	CACCATGGACACGCTTTGCTCCACRCTC	68
VH3_EXT1	CATGGAGTTgGGGCTGAGCTGGGTYTTCC	71.3
VH3_EXT2	CATGGAGTTgGGGCTGAGYTGGGTTTTCC	69.7
VH3_EXT3	CATGGAGTTTGGGCTGAGCTGGR	61.1
VH3_EXT4	CATGGAGTTTGGGCTGAGCTKGGTTTTYC	66.8
VH4_EXT1	ACCATGAAGCACCTGTGGTTCTBCCTCCTCC	69.7
VH4_EXT2	CACCATGAAGCACCTGKGGTTCTTY	60.1
VH5_EXT	CACCATGGGGTCAACTGCCMTCCTC	65.3
VH6_EXT	CCATGTCTGTCTCCTTCCTCATCGTCC	63.8
A_EXT	GAAGAAGCCCTGGACCAGGCAGGC	66.6
G-EXT	AAGGTGTGCACGCCGCTGGTCAG	66.9
M_EXT	GTCGGGAAGGAAGTCCTGTGCGAGG	66.5
D_EXT	TCCCCAGGTGCCAGGTGACAGTCAC	66.8
E_EXT	ACGGTCAGCAAGCTGATGGTGGCA	66.3

IgK primers		
VK1_EXT1	CACCATGGACATGAGGGYCCC	58.4
VK1_EXT2	CACCATGGACATGAGGGTCCCCAGTC	66.9
VK1_EXT3	CACCATGGACATGAGGGTCCCCGGTtAtC	70.2
VK1_EXT4	CACCATGGACATGAGGGTCYCCGGTCAG	69.5
VK1_EXT5	CACCATGGACATGAGGGTCCCCGGTCAGCTYC	75.9
VK2_EXT1	CACCATGAGGCTCCCWGCTCAG	60.6
VK2_EXT2	CACCATGAGGCTCCCTGCTCAGCTYCTGGGGC	76.8
VK3_EXT1	CACCATGGAAGCCCCAGCTCRGCTTCTC	69.7
VK3_EXT2	CACCATGGAAGCCCCAGCACAGCTTCTC	70.4
VK4_EXT	CACCATGGTGTCACAGACCCAAGWCTTC	64.4
VK5_EXT	CACCATGGGATCCCAGGTTCACCTCCTCAG	71.4
VK6_EXT1	CACCATGGTGTCCCCATTGCAACTCCTG	69.9
VK6_EXT2	CACCATGtTGTCtCCATCACAACTCATtG	63.2
VK7_EXT	CACCATGGGGTCCTGGGCTCCTTTCCTG	72.2
CK_EXT	ACCTGATCCTCAGATGGCGGGAAGATG	66.7
IgL primers		
VL1_EXT1	CACCATGGCCTGGTCTCCTCTCSTCCTCAC	69.7
VL1_EXT2	CACCATGGCCTGGTCTCCTCTCCTtCTC	67.4
VL2_EXT	CACCATGGCCTGGGCTCTGSTCCTC	67.2
VL3_EXT1	CACCATGGCCGGGACCCYTCTCCTCCTC	73.1
VL3_EXT2	CACCATGGCCTGGACCCCTGTTCTGCTC	71.8
VL3_EXT3	CACCATGGCCtGGACCCcTCcCCTRCTC	73.1
VL4_EXT	CACCATGGCCTGGACCCCACTCCTCCTC	72.6
VL5_EXT	CACCATGGCCTGGACTCYTCTC	57.3
VL6_EXT	CACCATGGCCTGGGCTCCACTCCTCCTC	72.7
VL7_EXT	CACCATGGCCTGGACTCTGCTCCTCCTCC	72
VL8_EXT	CACCATGGCCTGGATGATGCTTCTCCTCG	71.6
VL11_EXT	CACCATGGCCCTGACTCCTCTCCTCCTC	69.1
CL_EXT	TGCCATCTGCCTTCCAGGCCACTT	66.3

Primers	Sequences	Tm
PCR2 primers		

IgH primers		
VH1+7_INT1	CCAAGCTGGCTAGCACCATGGACTGGACCTGGAGGMTCCTC	80.1
VH1+7_INT2	CCAAGCTGGCTAGCACCATGGACCTSAcccGGAGSATCCTTTTC	78.4
VH2_INT	CCAAGCTGGCTAGCACCATGGACACGCTTTGCTCCAC	78.9
VH3_INT1	CCAAGCTGGCTAGCACCATGGAGTTGGGGCTGAGYTG	78.2
VH3_INT2	CCAAGCTGGCTAGCACCATGGAGTTTGGGCTGAGCTKG	78.5
VH4_INT	CCAAGCTGGCTAGCACCATGAAGCACCTGKGGTTC	75.3
VH5_INT	CCAAGCTGGCTAGCACCATGGGGTCAACTGCCMTCCTC	79
VH6_INT	CCAAGCTGGCTAGCACCATGTCTGTCTCCTTCCTCATCGTC	77.8
A_ACD_Int	CAGGGCCGCTGTGCTCTCGGAGGTGCTCCTGCCCTCGAGG CTCAGCGGGAAGAC	76.3
A_BC_Int	CAGGGCCGCTGTGCTCTCGGAGGTGCTCCTGCTGCAGAGG YTCAGCGGGAAGAC	75.5
G_Int	CAGGGCCGCTGTGCTCTCGGAGGTGCTCCTGGAG	80.9
M_Int	CAGGGCCGCTGTGCTCTCGGAGGTGCTCCTACAGGAGACG AGGGGGAAAAGGG	61.2
D_Int	CAGGGCCGCTGTGCTCTCGGAGGTGCTCCTCGCTGATATG ATGGGGAACACATCC	62.1
IgK primers		
VK1_INT1	CCAAGCTGGCTAGCACCATGGACATGAGGGYCCC	76.6
VK1_INT2	CCAAGCTGGCTAGCACCATGGACATGAGGGTCYCCG	78
VK2_INT1	CCAAGCTGGCTAGCACCATGAGGCTCCCWGCTC	75.8
VK3_INT1	CCAAGCTGGCTAGCACCATGGAAGCCCCAGCWC	76.7
VK4_INT	CCAAGCTGGCTAGCACCATGGTGTCACAGACCCAAG	76.4
VK5_INT	CCAAGCTGGCTAGCACCATGGGATCCCAGGTTCACCTCC	80.3
VK6_INT1	CCAAGCTGGCTAGCACCATGGTGTCCCCATTGCAACTC	79
VK6_INT2	CCAAGCTGGCTAGCACCATGTTGTCtCCATCACAACTC	74.9
VK7_INT	CCAAGCTGGCTAGCACCATGGGGTCCTGGGCTCCTTTCC	81.5
CK_INT	TGGCGGGAAGATGAAGACAGATGGTG	66.2
IgL primers		
VL1_INT	CCAAGCTGGCTAGCACCATGGCCTGGTCTCCTCTC	76.7
VL2_INT	CCAAGCTGGCTAGCACCATGGCCTGGGCTCTGSTCC	79.5
VL3_INT1	CCAAGCTGGCTAGCACCATGGCCGGGACCCYTC	78.8

VL3_INT2	CCAAGCTGGCTAGCACCATGGCCtGGACCCCTS	77.4
VL4_INT	CCAAGCTGGCTAGCACCATGGCCTGGACCCCACTCC	80.6
VL5_INT	CCAAGCTGGCTAGCACCATGGCCTGGACTCYTCTC	75.4
VL6_INT	CCAAGCTGGCTAGCACCATGGCCTGGGCTCCACTC	79.4
VL7_INT	CCAAGCTGGCTAGCACCATGGCCTGGACTCTGCTCCTC	79.5
VL8_INT	CCAAGCTGGCTAGCACCATGGCCTGGATGATGCTTCTC	78.6
VL11_INT	CCAAGCTGGCTAGCACCATGGCCCTGACTCCTCTCCTC	78.9
CL_INT	GTCACTGATCAGACACACTAGTGTGG	54.4
Sequencing primers		
VHKLSEQ_F	CCAAGCTGGCTAGCACCATG	54.7
VHSEQ_R	CTGTGCTCTCGGAGGTGCTCC	55.7
VKSEQ_R	GGAAGATGAAGACAGATGGTG	48.4
VLSEQ_R	TGATCAGACACACTAGTGTGG	45.1
Primers for the construction of linear cassettes		
Primers for P_{CMV} fragment[1]		
HV13220CMV-P-F262	AGTAATCAATTACGGGGTCATTAGTTCATAG	57.9
CMV-P_R	CATGGTGCTAGCCAGCTTGGGTC	60.6
Primers for Rh IgG1C fragment[2]		
RhIGC_HF	GGAGCACCTCCGAGAGCACAGC	62
BGH-R1235	TCCCCAGCATGCCTGCTATTGTC	61.9
Primers for Rh IgKC fragment[3]		
RhIGC_KF	CACCATCTGTCTTCATCTTCCCGCCA	65.7
BGH-R1235	TCCCCAGCATGCCTGCTATTGTC	61.9
Primers for Rh IgLC fragment[4]		
RhIGC_LF	CCACACTAGTGTGTCTGATCAGTG	51.3
BGH-R1235	TCCCCAGCATGCCTGCTATTGTC	61.9
Primers for overlapping PCR		
HV13220CMV-P-F262	AGTAATCAATTACGGGGTCATTAGTTCATAG	57.9
BGH-R1235	TCCCCAGCATGCCTGCTATTGTC	61.9

[1] pcDNA3.1 plasmid as template
[2] Plasmid with rhesus whole IgG1 gene as template
[3] Plasmid with rhesus whole IgK gene as template
[4] Plasmid with rhesus whole IgL gene as template

Supplementary Table 4   Statistical analysis Raw p values and false discovery rate p values.

Figure	Category	Test	Comparison	Variable	Compartment	Germline	Raw_P	fdr_p
Figure 2E	Homology	Wilcoxon	AGM:HU vs AGM:RM			V-Heavy	0.0000	0.0000
Figure 2E	Homology	Wilcoxon	AGM:HU vs AGM:RM			V-Kappa	0.0000	0.0000
Figure 2E	Homology	Wilcoxon	AGM:HU vs AGM:RM			V-Lambda	0.0000	0.0000
Figure 3B	Genomic	Fisher's Exact	HIV+/-: AGM vs HU		All		0.0000	0.0000
Figure 3B	Genomic	Fisher's Exact	HIV+/-: AGM vs RM		All		0.3095	0.5364
Figure 3B	Genomic	Fisher's Exact	HIV+/-: AGM vs HU		BM		0.0037	0.0113
Figure 3B	Genomic	Fisher's Exact	HIV+/-: AGM vs HU		PBMC		0.0000	0.0000
Figure 3B	Genomic	Fisher's Exact	HIV+/-: AGM vs RM		PBMC		1.0000	1.0000
Figure 3B	Genomic	Fisher's Exact	gp120+/-: AGM vs HU		All		1.0000	1.0000
Figure 3B	Genomic	Fisher's Exact	gp120+/-: AGM vs RM		All		0.0014	0.0052
Figure 3B	Genomic	Fisher's Exact	gp120+/-: AGM vs HU		BM		0.5403	0.7236
Figure 3B	Genomic	Fisher's Exact	gp120+/-: AGM vs HU		PBMC		0.4375	0.6319
Figure 3B	Genomic	Fisher's Exact	gp120+/-: AGM vs RM		PBMC		0.0004	0.0018
Figure 4	Genomic	Kolmogorov-Smirnov	ENV+ vs ENV-	HCDR3_Length			0.8996	1.0000
Figure 4	Genomic	Wilcoxon	ENV+ vs ENV-	H_Mutated			0.6547	0.7738
Figure 4	Genomic	Kolmogorov-Smirnov	gp120 vs Env cross-reactive	HCDR3_Length			0.3934	0.6016
Figure 4	Genomic	Exact Wilcoxon	gp120 vs Env cross-reactive	H_Mutated			0.5566	0.7236
Figure 4	Genomic	Kolmogorov-Smirnov	gp120 vs gp41	HCDR3_Length			0.9733	1.0000
Figure 4	Genomic	Exact Wilcoxon	gp120 vs gp41	H_Mutated			0.0401	0.0745
Figure 4	Genomic	Kolmogorov-Smirnov	gp41 vs Env cross-reactive	HCDR3_Length			0.6483	0.7738
Figure 4	Genomic	Exact Wilcoxon	gp41 vs Env cross-reactive	H_Mutated			0.3369	0.5475

Supplementary Table 5 % blocking by AGM gp120-specific ADCC mediating antibodies with undefined SIVsabgp120 fine-epitope specificity

% blocking				
	DH546	DH550	DH549	DH552
DH546-biotin	73.9	0	0	17
DH550-biotin	0	35.4	48.6	6.9
DH549-biotin	0	19.7	57.3	5.1
DH552-biotin	22.7	0	3.6	53.23

Supplementary Table 6 AGM and RM total B cell ($CD3^{-}$ $CD20^{+}$) and memory B cell ($CD20^{+}$ IgD^{-} $CD27^{+}$) count (cells/µl) at week 45 post infection

	animal ID	total B cell count (cells/µl) [1]	Mann-Whitney test	total memory B cell count (cells/µl) [2]	Mann-Whitney test
AGM	013-89-10	220	p = 0.07	118	p = 0.26
	013-90-10	21		8	
	013-91-10	36		11	
	013-92-10	116		59	
	013-93-10	12		4	
	013-94-10	149		83	
RM	206-96	289		109	
	257-97	95		40	
	402-98	662		376	
	403-98	251		66	

[1] denotes $CD3^{-}$ $CD20^{+}$ cells
[2] denotes $CD20^{+}$ IgD^{-} $CD27^{+}$
